# Copycat cannibals: witnessing cannibalism early in life affects adult behaviour

**DOI:** 10.1007/s00442-025-05748-7

**Published:** 2025-06-20

**Authors:** Ítalo Marcossi, Morgana M. Fonseca, Sarah F. J. Souza, Caio H. B. de Assis, Rafael S. Iasczczaki, Gabriel M. Beghelli, Rafael Bittencourt, Angelo Pallini, Yasuyuki Choh, Arne Janssen

**Affiliations:** 1 Department of Entomology, Federal University of Viçosa, Viçosa, Minas Gerais 36570-900 Brazil; 2 Agriculture and Livestock Research Enterprise of Minas Gerais/EPAMIG, Prudente de Moraes, MG Brazil; 3 Laboratory of Applied Entomology, Department of Horticulture, Chiba University, Chiba, Japan; 4 Evolutionary and Population Biology, IBED, University of Amsterdam, Science Park 904, 1098 XH Amsterdam, The Netherlands

**Keywords:** Ontogeny, Behaviour, Tinbergen’s why questions, Phytoseiids, Learning

## Abstract

Cannibalism is ubiquitous in the animal kingdom. The evolution and causation of cannibalistic behaviour have been amply investigated, but the ontogeny has received less attention. Here, we studied the ontogeny of cannibalistic behaviour in the tiny, blind predatory mite *Amblyseius herbicolus*. We found that individuals that were exposed to egg-cannibalizing adults when juvenile developed into cannibalistic adults more than 2.5 times as often as juveniles without such exposure. This was not due to their experience with eggs pierced by the adults: exposing juveniles to artificially pierced eggs did not result in increased cannibalism upon becoming adult. The exposure of juveniles to cannibalistic adults did not result in significant increases in juvenile mortality; hence, no selection against certain behavioural syndromes occurred during the juvenile stages. We therefore conclude that the experience with cannibalistic adults changed the behaviour of juveniles later in life. To the best of our knowledge, this is the first study showing that witnessing cannibalism as juvenile results in a higher tendency to cannibalize as adult.

## Introduction

During their development, prey may become invulnerable to predation, for example, because they simply become too big to be attacked by certain predators. This has been studied in detail for various aquatic organisms, which start life as potential victims, but outgrow the risk of being preyed upon by certain predators (Ferrari et al. 2019; Horn et al. 2019). However, this is only part of the possible effects of ontogeny. Small predators may be vulnerable to predation by large adult prey when small, but when large, later in life, become predators of these prey (Saito 1986; Polis et al. 1989; Polis 1991; Palomares and Caro 1999; Janssen et al. 2002; Magalhães et al. 2005). Thus, a reversal of the ecological roles of individuals can take place during ontogeny, where small individuals of species A are preyed upon by large individuals of species B, whereas large individuals of species A feed on individuals of species B. This especially holds for species involved in reciprocal intraguild predation (Montserrat et al. 2008; Marques et al. 2018; Fonseca et al. 2018).

Such role reversals imply that experience of a young individual with an individual of the other species may change its behaviour towards this other species later in life. For example, Choh et al. (2012) studied a system of two adult predatory mites engaged in intraguild predation of juveniles of the other species. They showed that individuals of one species that had been exposed to but escaped intraguild predation by the other species when juvenile, developed into adults that killed juveniles of this other species at a faster rate than unexposed juveniles. This shows that the effects of juvenile experience can affect behaviour of later developmental stages, and it can even affect behaviour after metamorphosis (Blackiston et al. 2008). However, there is more: the behaviour itself may also be subject to ontogenetic changes, not only because behaviour of different life stages differs, but also because the ecological role of individuals changes throughout development. In the case of Choh et al. (2012), the juvenile behaviour consisted of avoiding being attacked by the adults of the other species, but the individuals that survived subsequently actively hunted for the juveniles of the other species.

Similar changes in behaviour through ontogeny may occur with respect to cannibalism, where size or stage determines whether an individual is a victim or cannibal (Polis 1981; Elgar and Crespi 1992; Claessen et al. 2000; Persson et al. 2000). Cannibalism is a common ecological interaction in many animal taxa (Fox 1975; Polis 1981; Elgar and Crespi 1992; Richardson et al. 2010; Fouilloux et al. 2019). The proposed benefits of cannibalism include acquisition of nutrients (Fox 1975), exclusion of potential competitors for resources and mates, and elimination of a potential predator (Polis 1981; Elgar and Crespi 1992; DeVore et al. 2021). Costs associated with cannibalism are the possibility of infection with diseases and parasites, the loss of inclusive fitness when relatives are consumed, and the risks of being injured when attacking dangerous prey (Elgar and Crespi 1992; Pfennig 1997; Pfennig et al. 1998). Several ecological factors can promote the occurrence of cannibalism, but it often occurs as a response to low availability of high-quality food (Polis 1981). The incidence of cannibalism also varies with life history factors such as sex, age, kinship, and developmental asynchrony within populations (Hastings and Costantino 1991; Pfennig 1997; Bosch and Gabriel 1997; Schausberger and Croft 2001; Bayoumy and Michaud 2015; Revynthi et al. 2020). Cannibalism can have various effects on population dynamics and species persistence; it can prevent or cause extinctions of populations, can result in multiple dynamical attractors, including chaos, or may result in stabilizing or fluctuating dynamics (van den Bosch et al. 1988; Claessen et al. 2000, 2004; Miller and Rudolf 2011).

The reasons for occurrence of cannibalistic behaviour are often presented as a mixture of proximate and ultimate factors, i.e. a mixture of answers to several of the four “why” questions of Tinbergen (Tinbergen 1963; see also Krebs and Davies 1984), but, as Tinbergen argued, we need answers to all four questions for a proper understanding of behaviour. For example, the analysis of cannibalistic behaviour in terms of the costs and benefits mentioned above is an example of addressing the question of the adaptive value, and considering cannibalism as being genetically predisposed is an explanation in terms of the evolutionary history. However, this leaves questions of causation and the development of cannibalistic behaviour during ontogeny unanswered. This ontogeny is particularly important because individuals change from potential victims to potential cannibals during their lifetime. Here, we address this last question of ontogeny of cannibalistic behaviour, which, in our opinion, is somewhat underrepresented in the study of cannibalistic behaviour. We specifically investigate the role of juvenile experience with cannibalism on cannibalistic behaviour when adult, with the idea that experience early in life can have significant, lasting effects later in life (Ferrari et al. 2010).

A group of predators that is known for being cannibalistic are mites of the family Phytoseiidae (Schausberger 2003; Rahmani et al. 2009; Revynthi et al. 2018, 2020). As with predation in general, cannibalism in phytoseiid mites is generally asymmetrical with respect to the life stage and the size of the cannibal and the victim (Elgar and Crespi 1992; Fonseca et al. 2018), with eggs and juveniles usually being the victims and adults the cannibals. We investigated the effects of juvenile experience with cannibalizing adults on cannibalistic behaviour upon becoming adult. We used the omnivorous predatory mite *Amblyseius herbicolus* (Chant) (Acari: Phytoseiidae) to assess the effect of exposure of juveniles to cannibalism on the cannibalistic behaviour of these individuals when reaching adulthood.

## Material and methods

*Amblyseius herbicolus* reproduces through thelytokous parthenogenesis; hence, populations consist of females only (De Moraes and Mesa 1988). It is a generalist predator, abundant in several crops and it feeds on pollen and extrafloral nectar (Rodríguez-Cruz et al. 2017; Iasczczaki et al. 2025), and can prey on several pest species, such as whitefly immatures (Cavalcante et al. 2015; Xin and Zhang 2021; Cardoso et al. 2025), broad mites (Rodríguez-Cruz et al. 2013; Duarte et al. 2015), spider mites (Reis et al. 2007; Franco et al. 2010), the tomato russet mite (Marcossi et al. 2025), thrips (Lam et al. 2019), and the important citrus pest and disease vector *Diaphorina citri* (Kalile et al. 2021, 2023; Jimenez et al. 2021). Cannibalism in *A. herbicolus* has been reported repeatedly, and mainly consists of adults and juveniles piercing eggs and larvae with their mouthparts and feeding on their contents (Ferreira et al. 2008; Marcossi et al. 2020; Hou et al. 2022; Zhang and Zhang 2022a, b).

A rearing of the predatory mite *A. herbicolus* was started in 2016 with mites collected from tomato plants in vegetable gardens located in the urban and rural areas of Prados (Minas Gerais, Brazil) (Cardoso et al. 2025). The mites were reared on arenas made of PVC sheets (15 × 10 cm) on top of foam pads (*h* = 4 cm), which were kept in plastic trays (29 × 14 × 4 cm) filled with water. Cotton wool soaked in water was wrapped around the edges of the arenas to prevent the escape of mites and to provide water to the mites (van Rijn and Tanigoshi 1999). Several small pieces of tent-shaped PVC sheet were placed on the arenas to serve as shelters. A small piece of cotton wool was placed below each tent as an oviposition site, and cattail pollen (*Typha* sp.) was offered as food. Several arenas with predator rearings were started for the experiments described below and were kept in a climate-controlled room (25 ± 2 ºC, 70 ± 10% RH, 12:12 L:D).

**Fig. 1:**
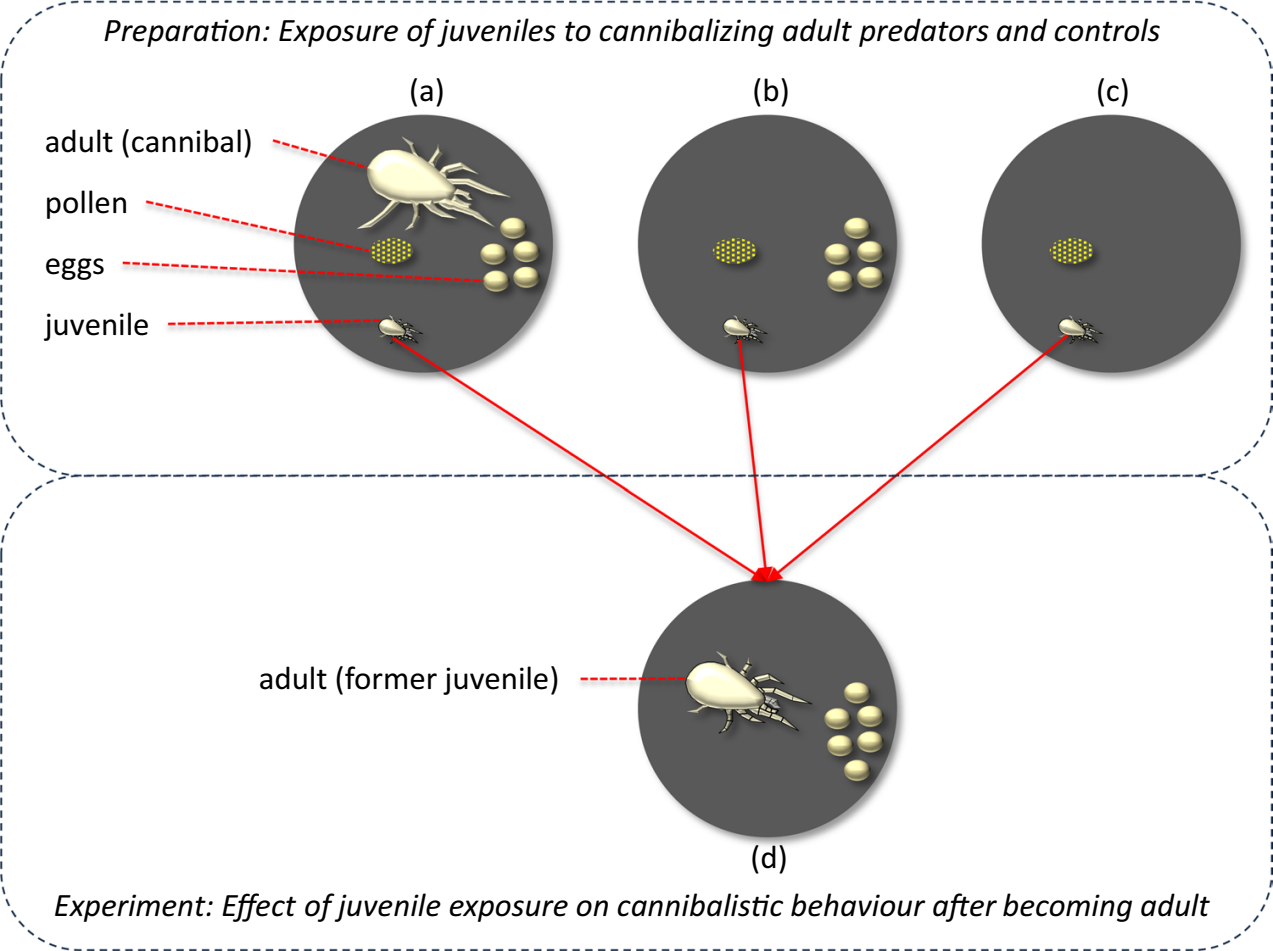
Experimental set-up of the preparatory phase (top) and the experimental phase (bottom). **a** Individual newly hatched larvae of *A. herbicolus* were incubated in a Petri dish (diameter 5 cm, only bottom shown here), one group with a cannibalizing adult conspecific, five conspecific eggs, and pollen as food. **b** A second group was similarly incubated, but without cannibalizing adult, and a third group **c** was incubated without an adult and eggs. In all cases (**a**–**c**), a small water-soaked piece of tissue paper was added as water source (not shown). Individuals were incubated until becoming adult. Eggs and pollen were renewed every day, but no pollen was supplied during one day. Survival and development of the juveniles and the numbers of cannibalized eggs were recorded every day. **d** After becoming adult, all individuals were individually given 6 eggs to cannibalize during one day, and the number of eggs cannibalized was recorded. Note that mites are not drawn to scale, the body (idiosoma) of adult females is c. 0.4 mm long, that of larvae is c. 170 µm (Ma et al. 2024)

Here, *A. herbicolus* eggs were used as victims of cannibals in all experiments (Marcossi et al. 2020). Experimental units consisted of plastic Petri dishes (*Ø* = 5 cm, *h* = 1.5 cm) closed with transparent lids, the bottom of the Petri dish was black to allow better observation of the mite eggs. All adults used in the experiments were gravid females, aged between 10 and 15 days after egg hatch. These females are less than 0.5 mm in size (Fig. 1). Adult females that were used to expose juveniles to cannibalism throughout their development were placed singly in the experimental units and were starved for 24 h before the start of the experiments.

To obtain the numbers of eggs needed to set up the experiment, 250 females were individually placed in experimental units. Each Petri dish contained a small piece of tissue paper soaked in water as a water source and a large amount of *Typha* sp. pollen as a food source. Eggs were collected and the food source replaced daily.

### Experimental design

We performed two experiments, which both consisted of a preparatory phase where juveniles were exposed (Fig. 1a–c), and an experimental phase where the cannibalistic behaviour of the exposed and unexposed individuals was tested after they became adult (Fig. 1d). In the first experiment, juveniles were exposed to cannibalizing adults, whereas we mimicked cannibalism in the second experiment by manually piercing eggs. Follows a description of both phases for the first experiment and a short description of the second experiment, in as far as it differed from the first experiment.

#### Exposure of juveniles to cannibalizing adult predators

Newly hatched larvae (less than 24 h old) of *A. herbicolus* were collected from the rearing arenas and each individual was placed in a similar Petri dish as above. To expose the larvae to the occurrence of cannibalism during their entire ontogeny, five *A. herbicolus* eggs (less than 24 h old) were taken from an arena and were added to the Petri dish, serving as victims for the cannibals. Care was taken to take the eggs from another arena as from which the juveniles were taken. A single starved adult female of *A. herbicolus* from another arena as the eggs and juveniles, and referred to as “cannibal”, was placed together with the larvae (Fig. 1a). It was unknown whether these females had cannibalized before. Notice that the cannibals could oviposit in the Petri dishes. To discriminate between eggs already present and eggs produced by the cannibal, we placed the eggs in a fixed pattern at the centre of the Petri dish. Ample cattail pollen (*Typha* sp., c. 0.5 mg) was provided and served as food, allowing both stages to feed freely on it (Fig. 1a). Water was provided by adding a small water-soaked piece of tissue paper. To prevent the presence of more larvae due the hatching of the eggs, all empty eggshells, non-cannibalized eggs, and those laid by the cannibal were removed with a fine brush every day, five new eggs were added, and pollen was also replaced. Because cannibalism by *A. herbicolus* is reduced in the presence of cattail pollen, which is a high-quality food source (Marcossi et al. 2020), no pollen was provided during the third day of exposure of the juveniles to stimulate cannibalism, but was provided again after that. This temporary lack of food may have delayed the development of the juveniles into adults. The juveniles were kept on these arenas for five days, but the adult predator was removed at the end of the fourth day to avoid confusion with the juveniles that were developing into adults. Two juvenile control groups were set up in a similar manner, but without adult cannibals. Hence, these individuals did not witness cannibalism by an adult during their development. The first control group was supplied with eggs from *A. herbicolus* plus pollen and water as above (Fig. 1b), and served to assess the incidence of cannibalism by the developing juvenile. The second control group was supplied with pollen and water, but without eggs (Fig. 1c); hence, juveniles could not cannibalize eggs while developing into adults. In both controls, no pollen was supplied on the third day as above.

To verify whether the risk of predation to which the prey was exposed affected the development until adulthood, we evaluated the development rate and juvenile survival in all groups. We furthermore recorded the number of cannibalized eggs daily to check for the occurrence of cannibalism. Cannibalized eggs were recognized as empty shells from which the content was removed (Yao and Chant 1989; Marcossi et al. 2020) and were observed with a stereoscopic microscope (Zeiss Stemi 2000-c). Twenty replicates were carried out for each treatment.

Data from the developmental rate and survival of the juveniles during their exposure were analysed with a time-to-event analysis (Cox proportional hazards model) using the function “coxph” of the “survival” package (Therneau 2020) in R (R Core Team 2023). To compare the occurrence of egg cannibalism in the presence or absence of the adult cannibal (i.e. comparing egg cannibalism by the adult plus juvenile with that by the juvenile alone), a generalized linear model with a binomial error distribution (logit link) was used (Crawley 2013).

#### Effect of juvenile experience on cannibalistic behaviour after becoming adult

We subsequently isolated individuals that had become adult from each treatment group above in an experimental unit (Petri dish) as above. Six eggs (less than 24 h old) from conspecific females from another arena were added to each dish. No other food was provided; hence, the adults could only feed on the eggs (Fig. 1d). The numbers of cannibalized eggs were assessed after 24 h using a stereoscopic microscope (as above).

To assess whether juveniles that developed into adults while exposed to cannibalizing conspecific adults engaged more often or less in cannibalism, we performed several analyses. Because the data were zero-inflated, we first compared the numbers of individuals that cannibalized or not among the three treatment groups with a GLM with a binomial error distribution (logit link, logistic regression). Not all juveniles that were exposed to adults experienced cannibalism, and some juveniles cannibalized eggs without adults being present; hence, juveniles from the two groups that had developed in the presence of eggs could be divided into a group with experience with cannibalism, either by an adult or by themselves, and a group that did not experience cannibalism. We subsequently compared the occurrence of cannibalistic behaviour of individuals of these two groups using a similar analysis with treatment and occurrence of cannibalism during development and their interaction as factors. The numbers of eggs cannibalized by those individuals that did cannibalize during the actual test of cannibalistic behaviour were compared with a generalized linear model (GLM) with a Poisson or quasi-Poisson model (in case of overdispersion) with treatment as fixed factor (log link). Contrasts among treatments (Tukey method) were assessed with the emmeans function of the package with the same name (Lenth 2019). All statistical analyses were performed with the software R 3.6.1 (R Core Team 2023).

#### Exposure of juveniles to artificially pierced eggs

To discriminate between experience with cannibalism by adult conspecifics and experience with pierced eggs, we exposed individuals to cannibalizing adults during their juvenile development as above (Fig. 1a) and exposed another group to eggs of which one egg was manually pierced every day without a cannibalizing adult conspecific being present. Eggs were pierced with a thin syringe needle. The cannibalizing adults in the experiment above killed on average 0.61 (s.e. 0.12) egg per day, so slightly more eggs were killed artificially during this exposure than were cannibalized during exposure in the previous experiment. As control, a group of individuals developed in the presence of eggs that were not pierced (Fig. 1c). The experimental procedure was the same as above, pollen was supplied on all days except for the third day. The developmental rate and juvenile survival until adulthood were measured and tested as above. The cannibalistic behaviour of the individuals that became adult was assessed as above.

## Results

### Effect of juvenile experience on cannibalistic behaviour later in life

**Fig. 2:**
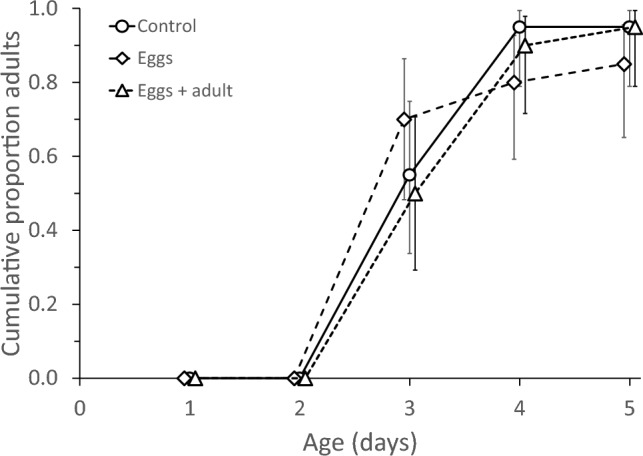
Development and survival of *Amblyseius herbicolus* juveniles that had either been exposed to adult cannibals when juvenile (“Eggs + adult”, Fig. 1a) or not (“Eggs”, Fig. 1b, and “Control”, Fig. 1c). Shown are the mean cumulative proportions of individuals that reached adulthood as a function of time. Error bars are 95% confidence intervals of proportions. Survival is given by the final proportion of adults (day 5). Development and survival did not differ significantly among treatments. Notice that data points are slightly jittered horizontally, but were assessed at the same time

Juvenile development and survival during the exposure period did not differ significantly among the three treatment groups (Fig. 2, Cox proportional hazards, development: log-rank test = 0.14, d.f. = 2, *P* = 0.9, survival: log-rank test = 1.79, d.f. = 2, *P* = 0.4). In the treatment in which the juveniles were exposed to the adult predator (Fig. 1a), only one juvenile was cannibalized. Among the 19 survivors, 15 (78.9%) juveniles experienced egg cannibalism during their development, either by the adult or by themselves. In the control treatment where juveniles had access to eggs (Fig. 1b), three juveniles (17.6%) cannibalized conspecific eggs. The incidence of egg cannibalism between these two groups was significant (GLM, deviance = 18.4, d.f. = 1, *P* < 0.0001).

**Fig. 3:**
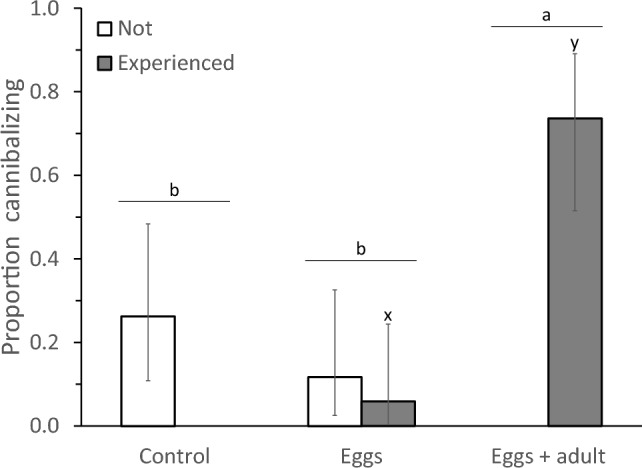
The proportions of cannibalizing adult females of *Amblyseius herbicolus* in the set-up shown in Fig. 1d, after they had either been exposed to adult cannibals when juvenile (“Eggs + adult”, Fig. 1a) or not (“Eggs”, Fig. 1b; and “Control”, Fig. 1c). “Experienced” shows the proportion of females that had witnessed cannibalism by an adult female (treatment “Eggs + adult”, grey bar) and/or had themselves cannibalized when juvenile (treatment “Eggs”, grey bar). White bars: individuals had neither witnessed nor engaged in cannibalism. Error bars are 95% confidence intervals of proportions (Jeffrey method). Letters (a, b) above the pairs of bars indicate significant differences among treatments, above grey bars (x, y) significant difference between the proportions of experienced individuals (contrasts after GLM)

Juvenile experience significantly affected the tendency to cannibalize after becoming adult (Fig. 3, GLM: deviance = 14.4, d.f. = 2, *P* = 0.0008). Individuals that were exposed to adult cannibals as juveniles (Fig. 1a) were more than 2.5 times more often cannibalistic later in life than individuals that were not exposed (Fig. 1c) or exposed to eggs only (Fig. 1b) (Fig. 3). Comparison of the two groups that had been exposed to eggs when juvenile (Fig. 1a and b) showed that the tendency to cannibalize as adult depended both on the presence of adults and on the occurrence of cannibalism during development (GLM, interaction between occurrence of cannibalism and presence of adult: deviance = 4.67, d.f. = 1, *P* = 0.031, Fig. 3). There was no significant difference in the tendency to cannibalize later in life among individuals that had not witnessed cannibalism and had not cannibalized as juvenile, but juveniles that had witnessed cannibalism in the presence of an adult became cannibalistic more often than individuals that had cannibalized without an adult being present (Fig. 3). When the adult present during the development did not cannibalize, the exposed individuals never cannibalized after becoming adult (Fig. 3). In the group of individuals that had been exposed to eggs only (Fig. 1b), one of the three individuals that had cannibalized when juvenile also cannibalized when adult, and two of the other 14 individuals cannibalized (Fig. 3).

**Fig. 4:**
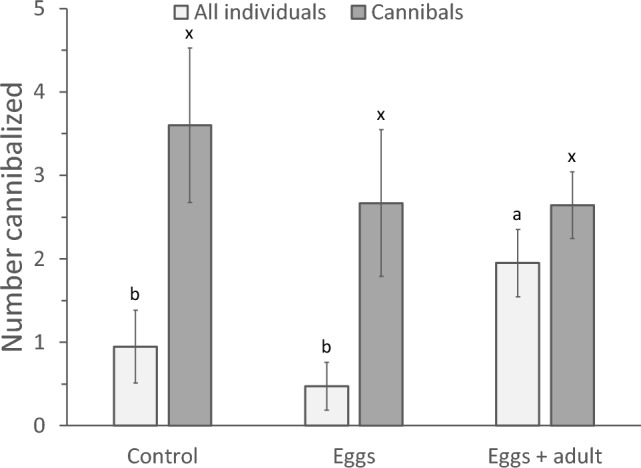
The average numbers of eggs cannibalized (± s.e.) per individual, which had either been exposed to adult cannibals when juvenile (“Eggs + adult”, Fig. 1a) or not (“Eggs”, Fig. 1b; and “Control”, Fig. 1c). Light bars show numbers of eggs cannibalized averaged over all individuals, dark bars are the number of eggs cannibalized averaged over those individuals that did cannibalize. Letters above error bars indicate significant differences among treatments (contrasts after GLM)

The numbers of eggs killed averaged over all individuals differed significantly with previous experience (Fig. 4, light bars, GLM, *F*_2,52_ = 3.29, *P* = 0.045), with individuals growing up with adults and eggs cannibalizing more than individuals of the other two treatments. However, there was no difference in the average numbers of eggs cannibalized by those individuals of the three treatments that did cannibalize (Fig. 4, dark bars, GLM, *F*_2,19_ = 0.627, *P* = 0.54). This shows that the experience with cannibalizing adults during juvenile development increased the propensity to cannibalize, but not the number of eggs cannibalized.

### Effect of pierced eggs and cannibalism

**Fig. 5:**
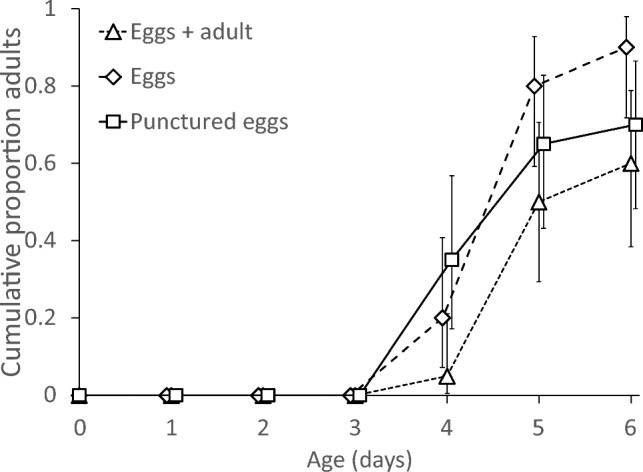
Development and survival of *Amblyseius herbicolus* juveniles that had either been exposed to adult cannibals when juvenile (“Eggs + adult”, as in Fig. 1a), to intact eggs (“Eggs”, as in Fig. 1c) or to eggs plus punctured eggs (“Punctured eggs”). Shown are the mean cumulative proportions (± 95% CI) of individuals that reached adulthood as a function of time. Survival is given by the final proportion of adults (day 6). Development and survival did not differ significantly among treatments. Notice that data points are slightly jittered horizontally, but were assessed at the same time

The survival and development of juveniles that were exposed to cannibalizing adults, to pierced eggs or intact eggs did not differ significantly among treatments (Fig. 5, Cox proportional hazards, development: log-rank test = 4.62, d.f. = 2, *P* = 0.1, survival: log-rank test = 4.81, d.f. = 2, *P* = 0.09). For unknown reasons, survival was lower than during the first experiment (cf. Figure 5 and 2).

**Fig. 6:**
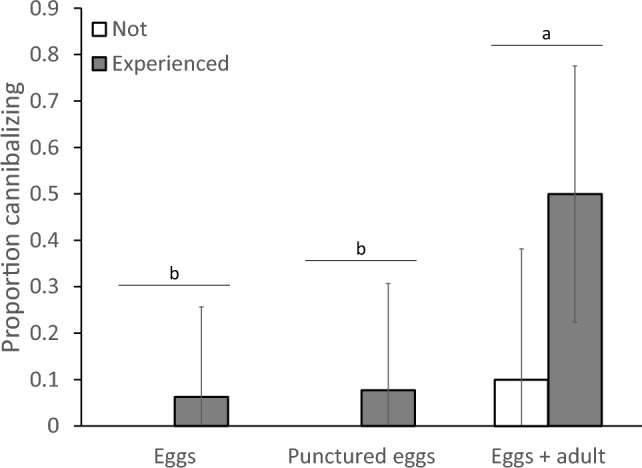
The proportion of cannibalizing adult females of *Amblyseius herbicolus* after they had either been exposed to adult cannibals when juvenile (“Eggs + adult”, as in Fig. 1a), to intact eggs (“Eggs”, as in Fig. 1c) or to eggs plus punctured eggs (“Punctured eggs”). “Experienced” shows the proportion of females that had themselves cannibalized when juvenile (treatment “Eggs”, grey bar) and/or had witnessed cannibalism by an adult female (treatment “Eggs + adult”, grey bar). White bars: individuals had neither witnessed nor engaged in cannibalism when juvenile. Error bars are 95% confidence intervals of total proportions (so of grey plus white bar of treatment “Eggs + adults”). Letters above the error bars indicate significant differences among treatments (contrasts after GLM)

The experience during juvenile development again significantly affected the probability to turn into a cannibalizing adult (GLM, deviance = 11.6, d.f. = 2, *P* = 0.003), with individuals that had been exposed to adults and eggs again cannibalizing significantly more than individuals that were exposed to eggs alone or to eggs plus punctured eggs (Fig. 6). Individuals that had not cannibalized when juvenile and had no experience with cannibalism in the presence of an adult had a low probability of cannibalizing later in life (Fig. 6, white bar).

**Fig. 7:**
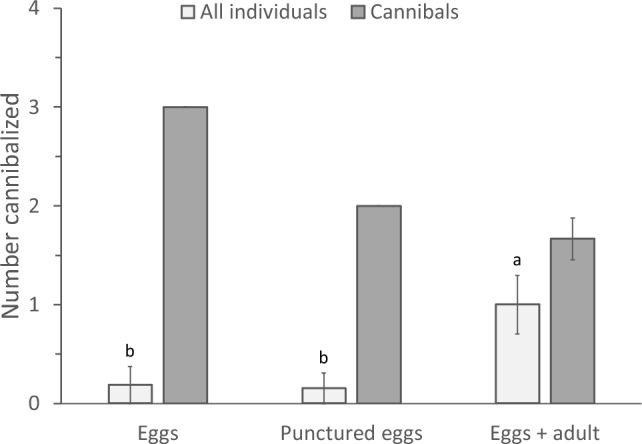
The average numbers of eggs cannibalized (± s.e.) per individual, which had either been exposed to adult cannibals when juvenile (“Eggs + adult”, as in Fig. 1a), to intact eggs (“Eggs”, as in Fig. 1a) or to eggs plus punctured eggs (“Punctured eggs”). Light bars show numbers of eggs cannibalized averaged over all individuals, dark bars the number of eggs cannibalized averaged over those individuals that did cannibalize. Letters above error bars indicate significant differences among treatments (contrasts after GLM)

The numbers of killed eggs averaged over all individuals again differed significantly with previous experience (GLM, deviance = 11.1, d.f. = 2, *P* = 0.004), with individuals growing up with adults and eggs cannibalizing more than individuals with the other two treatments (Fig. 7, light bars). Only one of the individuals growing up in the presence of eggs and one in the presence of punctured eggs cannibalized and we therefore did not perform a statistical test comparing the numbers of eggs attacked per cannibalizing adult among the tree treatment groups (Fig. 7). Together, these data show that the experience with punctured eggs does not increase the propensity to cannibalize.

## Discussion

In this study, we specifically addressed the ontogeny of cannibalistic behaviour, showing that individuals that were exposed to adult cannibals while juvenile developed more frequently into cannibalizing adults than unexposed individuals. Furthermore, juveniles that were only exposed to eggs did not develop into cannibalistic individuals more frequently than individuals that were not exposed to eggs, showing that the presence of adults during the juvenile period was the main determinant for an increase in the incidence of cannibalism later in life (Fig. 3). Moreover, it was not only the presence of an adult during their development that changed the cannibalistic behaviour, but also the occurrence of cannibalism that resulted in an increase in cannibalism later in life (Fig. 3, 6). This demonstrates that juvenile experience with cannibalistic adults induced a behavioural change in these individuals that was manifested upon becoming adult, suggesting that juveniles learned to cannibalize eggs from adults.

It is unclear how the juveniles learned to cannibalize. Because the mites are blind, visual cues such as seeing the adults attack and feed on eggs can be excluded. In a preliminary attempt to discover possible interactions between the adult and juvenile near the eggs, we recorded over 49 h of video footage of four pairs of adults and juveniles and observed cannibalism of eggs by adults and juveniles, but no physical interaction between the two individuals near the eggs. This suggests that physical interactions between adults and juveniles did not play a role in learning to cannibalize. Mites of the order Mesostigmata, which includes the Phytoseiidae family, are known to produce salivary secretions for extraoral digestion (Evans 1992). If this is the case for *A. herbicolus*, juveniles may recognize the enzymes released when cannibalistic adults feed on eggs and may associate this with food. Although we did not observe this in the videos, the juveniles could have scavenged on the remains of the eggs damaged by adults and associated the presence of eggs with the availability of food. This association then could have resulted in the recognition of eggs as food later in life. Juveniles could also have scavenged on the artificially pierced eggs offered to them in the second experiment, but if they did, this did not result in an increased inclination to cannibalize later in life (Fig. 6).

We specifically measured and reported the juvenile survival of the individuals during the preparatory phase of both experiments. This is important with this type of experiments because it serves to rule out the possibility of selective mortality (Choh et al. 2012). Showing that the individuals surviving an exposure subsequently show other behaviour than a control group does not necessarily mean that they changed their behaviour as a result of exposure, but can be a result of variation in behaviour in the entire population and subsequent selective mortality of individuals with some types of behaviour. In our experiments, exposing juveniles to cannibalism could have resulted in some individuals falling victim while others survived. These survivors may then be inherently better at avoiding cannibalism than the victims, for example, because they are better at hiding, escaping, or counterattacking. This behaviour may be part of a behavioural syndrome (Sih et al. 2004), related to differences in behaviour later in life. The increased adult cannibalism may then be the result of selective survival of cannibalistic individuals, and not of behavioural changes in individuals that were exposed to a risk of cannibalism. In our first experiment, there was no such selection: survival was high for all treatments (Fig. 2). In the second experiment, however, survival was lower and lowest for juveniles exposed to cannibalizing adults, although not significantly so (Fig. 5). Thus, possibly, juveniles that died when exposed to cannibalizing adults may have been those that would cannibalize less when adult. Earlier, we introduced a method to estimate whether this selection would significantly affect results (Choh et al. 2012). In short, this comes down to removing those individuals that cannibalized less from the control groups, as if they were selected against by being cannibalized during the preparatory phase, and then testing the remaining individuals against those of the exposed group. Here, we used the same method and removed the same numbers of individuals from the control groups as died in the group exposed to adult cannibals during their preparatory phase by selecting individuals that did not cannibalize when adult. With these individuals removed, cannibalism in the group that had been exposed to adult cannibals was still significantly higher than that in the two control groups (GLM, deviance = 9.87, d.f. = 2, *P* = 0.007) (results not shown). We therefore conclude that selective mortality during the preparatory phase did not play an important role; hence, individuals changed their cannibalistic behaviour due to their juvenile experience with cannibalism by adults.

Several studies have documented the effects of experience early in life on behaviour later (Johnson 2005; Ferrari et al. 2019), also in predatory mites (Schausberger 2004; Schausberger et al. 2010). Rahmani et al. (2009) showed that adult females of the predatory mite *Phytoseiulus persimilis* cannibalized conspecific larvae quicker after having been allowed to cannibalize when juvenile, but females without experience with conspecific larvae also cannibalized, but only a few hours later. The differences described here were much larger: not just the speed of cannibalizing, but the actual tendency to cannibalize differed significantly with experience. To the best of our knowledge, this is the first study showing that witnessing cannibalism as juvenile results in a higher tendency to cannibalize as adult.

One important question is whether this cannibalistic behaviour is typical for laboratory strains or also occurs in the field. Rearing animals in the laboratory generally results in changes in behaviour, life history, and genetic variation (Mackauer 1976; Hopper et al. 1993), and cannibalism may be one of the behaviours that changes in the laboratory, mainly related to the densities in the rearing units (Dennehy et al. 2001). We showed earlier that adults of the strain of *A. herbicolus* used here did not cannibalize when high-quality food (*Typha* pollen) was present (Marcossi et al. 2020), and the cultures in our lab were fed ample amounts of this pollen. Possibly, when predator densities in the rearing units are high, there are short periods of limited food supply and there may be strong selection for cannibalism during these periods (Dennehy et al. 2001; Revynthi et al. 2018). Based on our results, we expect that juveniles that have witnessed cannibalism in the laboratory cultures will be more cannibalistic when adult. It is therefore important to maintain cultures with ample amounts of good-quality food to avoid cannibalism (Vangansbeke et al. 2014). Under more natural conditions, predators may choose not to cannibalize but to disperse to a new patch with resources (Revynthi et al. 2020). Future research could assess whether laboratory strains of *A. herbicolus* have a higher tendency to cannibalize than strains in the field, where they can disperse. Furthermore, several species of phytoseiid mites, including the parthenogenetic *A. herbicolus*, can distinguish unrelated from related conspecifics (i.e. kin recognition) (Faraji et al. 2000; Schausberger and Croft 2001; Zhang and Zhang 2022a, b). It should be noted that the individuals tested here were not kin-related to the eggs or the cannibals with which they gained experience and were tested. It would be interesting to investigate if exposure of *A. herbicolus* juveniles to cannibalism of kin eggs or to kin adult cannibals changes their cannibalistic behaviour later in life.

The four “why” questions on behaviour of Tinbergen (1963) are all important for a proper understanding of behaviour. The predatory mites used here all share the same evolutionary history, and cannibalism occurs in many animal species (Polis 1981), including many predatory mite species (Schausberger 2003). It is furthermore well established that the presence of high-quality food decreases cannibalism (Polis 1981; Montserrat et al. 2006); also in *A. herbicolus*, the predatory mite investigated here (Marcossi et al. 2020), suggesting that the function or adaptive value is to obtain food and/or reduce future intraspecific competition. Lack of high-quality food may also be the proximate trigger causing cannibalism. Despite the similarities in evolutionary history, function, and causation among the groups tested here, there were large differences in cannibalistic behaviour among them, showing the importance of the ontogenetic development of this behaviour. Because of its relevance for population dynamics and species persistence (van den Bosch et al. 1988; Claessen et al. 2000, 2004; Miller and Rudolf 2011), it is important to study all aspects of cannibalism, including its ontogeny.

## Data Availability

Data are available on 10.21942/uva.29375891.v1.
